# Can KOOS-PS be replaced with a simple anchor question in patients after total knee arthroplasty?: an agreement study of 2,478 primary surgeries

**DOI:** 10.2340/17453674.2024.42098

**Published:** 2024-11-12

**Authors:** Siri B WINTHER, Anders SJØSTRØM, Sølvi LIABAKK-SELLI, Olav A FOSS, Tina S WIK, Jomar KLAKSVIK

**Affiliations:** 1 Department of Orthopedic Surgery, St. Olav’s Hospital HF, Trondheim; 2 Department of Neuromedicine and Movement Science, Faculty of Medicine and Health Science, Norwegian University of Science and Technology NTNU, Trondheim, Norway

## Abstract

**Background and purpose:**

Physical function and pain are the most important outcomes following total knee arthroplasty (TKA). These can be evaluated by patient-reported outcome measures (PROMs), or by an anchor question. The primary aim of the study was to evaluate whether a simple anchor question can replace KOOS-PS in assessing postoperative knee function until 1-year follow-up, evaluated by analyzing the agreement between the 2 methods using the diagnostic odds ratio (DOR). Secondary aims were pain (NRS) at rest and during mobilization.

**Methods:**

This is a diagnostic accuracy study with primary TKAs performed between 2010 and 2022. The surgeries were categorized as improved (I) or worsened (W) based on a dichotomized anchor question related to self-perceived change in physical function, and the dichotomized change in KOOS-PS until 1-year follow-up. This led to 4 groups: (II, IW, WI, and WW).

**Results:**

Agreement was found with a DOR of 11.3 (CI 7.9–16.2). 2,335 (94%) reported improved function on the anchor question and 143 (6%) worsened function. Among those with improved anchor 2,132 (91%) had improved KOOS-PS, but among those with worsened anchor only 74 (52%) had worsened KOOS-PS. Pain at 1-year follow-up was lower in the groups reporting improved anchor.

**Conclusion:**

The KOOS-PS can be replaced with an anchor question to assess change in function until 1 year. However, the KOOS-PS might be a valuable supplement in patients reporting worsened anchor as only half of those had worsened KOOS-PS.

Physical function and pain are the most important outcomes following total knee arthroplasty (TKA), and the patient’s evaluation of a successful outcome depends on the fulfillment of their preoperative expectations related to these outcomes [1-4]. Patient-reported outcome measures (PROMs) have increasingly been used to evaluate postoperative outcomes following orthopedic surgery [5]. However, it is speculated that there is only a weak to moderate correlation between disease-specific PROMs and patient satisfaction, suggesting that a single PROM is not sufficient for this purpose [6].

Alternatively, an anchor question can be used postoperatively to evaluate the patient’s opinion on the outcomes. Patients either rate their satisfaction related to general health, pain, or physical function derived from validated questionnaires [7], or based on self-designed questions related to specific outcomes and their postoperative relative to preoperative status [8,9]. Our research group has previously demonstrated that 6% of all primary TKAs in an institutional registry reported worsened joint function based on a 1-year anchor question [10]. However, studies published on retrospective data collection have shown that the patients’ ability to recall their preoperative pain and physical function is not accurate at 1 year following TKA [11-13].

Ideally, if the patient’s response to the anchor question was able to reveal the true change in knee function, the response should be consistent with the change in the disease specific PROM questionnaire, the Knee Injury and Osteoarthritis Outcome Score – Physical Function – Short Form (KOOS-PS) [14] from preoperatively to 1-year follow-up. The aim of the study was therefore to evaluate whether a simple anchor question can replace KOOS-PS in assessing postoperative knee function until 1-year follow-up after primary TKA. We hypothesized that there is an agreement between the anchor question and the KOOS-PS.

## Methods

### Design

A diagnostic accuracy study was undertaken with prospectively recorded data from an institutional registry for patients with TKA. All patients followed the standardized fast-track clinical course at our orthopedic department, which emphasizes extensive preoperative information concerning all parts of the treatment, including expected postoperative results. Furthermore, all patients are provided the same postoperative regime with a focus on early mobilization and mobility, and encouraged to be physically active. Further details of the fast-track clinical pathway have been described previously [15]. The study is reported according to STARD and GRRAS guidelines.

### Patients

**Figure 1:**
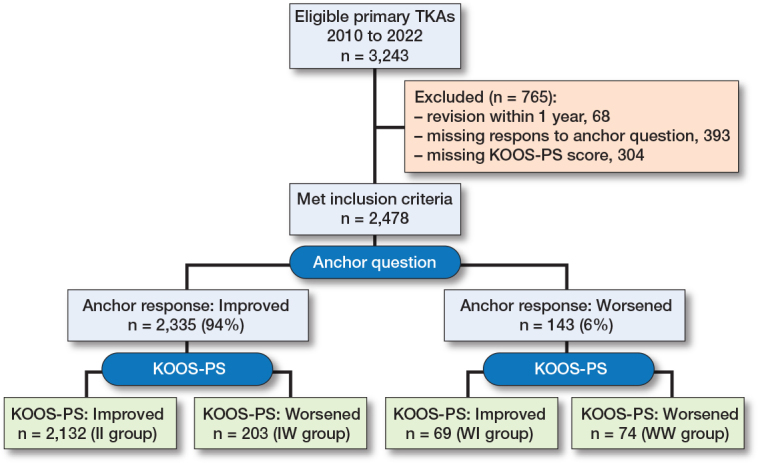
Patient flowchart. KOOS-PS = Knee Injury and Osteoarthritis Outcome Score – Physical Function, TKA = total knee arthroplasty.

All elective primary TKAs performed at St. Olav’s University Hospital between 2010 and 2022, attending 1-year follow-up, were eligible for study participation. Exclusion criteria were revision within 1 year, missing data on the anchor question related to self-perceived knee function at 1-year follow-up, and the KOOS-PS questionnaire, which assesses physical function. A flowchart of patient enrollment is presented in Figure 1.

### Outcomes

The KOOS-PS ranges from 0 to 100, the latter representing no difficulty in the performance of specific tasks [14]. The anchor question, “How is the function of the operated on joint today compared with before surgery?,” has 4 possible responses where the patients rate their knee function as “better,” “same,” “unable to discriminate,” or “worse,” relative to their recall of preoperative function. These responses were dichotomized with “worse” defined as “worsened” and the rest as “improved”. Accordingly, the changes in KOOS-PS from preoperatively to 1-year follow-up were dichotomized with negative changes (Δ < 0) defined as “worsened” and positive changes (Δ ≥ 0) as “improved”.

We analyzed the agreement between the anchor question and KOOS-PS using the diagnostic odds ratio (DOR) with 95% confidence intervals (CI) as the primary endpoint. Secondary outcomes were pain during mobilization and pain at rest, reported by the numeric rating scale (NRS 0–10) (0 representing no pain). Surgery time, length of hospital stay (LOS), and postoperative complications were also registered.

**Figure 2:**
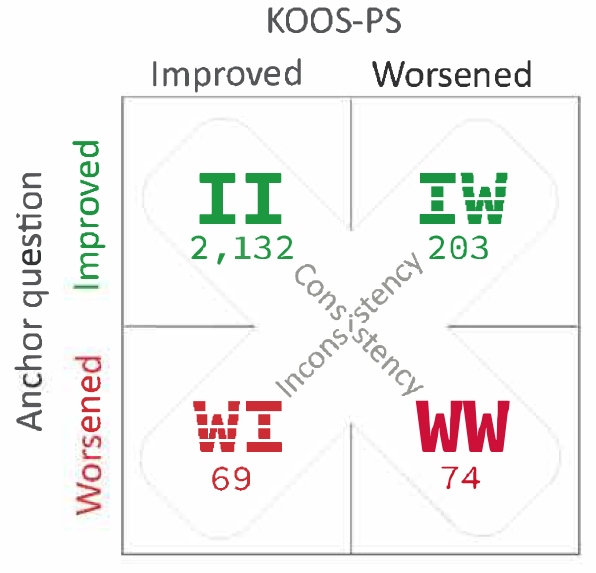
Groups dichotomized by the anchor question, and accordingly change in KOOS-PS. WW = worsened anchor/worsened KOOS-PS, WI = worsened anchor/improved KOOS-PS, IW = improved anchor/worsened KOOS-PS, II = improved anchor/improved KOOS-PS.

The patients were divided into 4 groups: (i) improved anchor and improved KOOS-PS (II), (ii) worsened anchor and worsened KOOS-PS (WW), (iii) improved anchor and worsened KOOS-PS (IW), and (iv) worsened anchor and improved KOOS-PS (WI) (Figure 2). Patient demographics are presented in the Table.

**Table:** Patient demographics and clinical outcomes. Values are presented as mean (range) or n (%)

Factor	WW (n = 74)	WI (n = 203)	IW (n = 2,132)	II (n = 2,478)	Total
Age	64 (45–84)	66 (41–87)	67 (41–91)	67 (22–92)	67 (22–92)
Body mass index	29 (17–42)	30 (18–42)	29 (15–57)	29 (15–51)	29 (15–57)
Female sex	45 (61)	45 (65)	115 (57)	1,346 (63)	1,551 (63)
ASA					
I	10	7	34	263	314
II	49	48	122	1,361	1,580
III	14	14	45	494	567
IV	0	0	1	10	11
Surgery time (min)	86 (45–136)	92 (56–183)	88 (50–162)	89 (47–235)	89 (45–235)
LOS (days)	2.7 (1–7)	2.8 (1–14)	2.8 (1–9)	2.6 (0–12)	2.6 (0–14)
Complications^a^					
Deep infection	0	1 (1.4)	1 (0.5)	7 (0.3)	9 (0.4)
Mechanical	1 (1.4)	0	2 (1.0)	0	3 (0.1)
Stiffness	15 (20)	9 (13)	8 (3.9)	75 (3.5)	107 (4.3)
DVT	2 (2.7)	0	3 (1.5)	10 (4.7)	15 (0.6)
Readmission^b^	6 (8.1)	0	4 (2.0)	36 (1.7)	46 (1.9)
Reoperation^c^	9 (11)	10 (14)	3 (1.5)	27 (1.3)	48 (1.9)

ASA = American Society of Anesthesiologists physical status, LOS = length of hospital stay, DVT = deep vein thrombosis, WW = worsened anchor/worsened KOOS-PS, WI = worsened anchor/improved KOOS-PS, IW = improved anchor/worsened KOOS-PS, II = improved anchor/improved KOOS-PS.

a 1 surgery could be registered with several complications.

b Readmissions without any reoperations.

c Revisions within 1-year follow-up.

### Data collection and outcomes

Data were prospectively collected by nurses, physiotherapists, and self-registered by the patients. Registration was performed at the preoperative outpatient clinic, during hospitalization, and twice after discharge; at 2-month and 1-year follow-up.

### Statistics

A DOR with CI was used to determine agreement between the dichotomized response on the anchor question and the dichotomized change in KOOS-PS (reference standard). The DOR ranges from 0 to infinity, with higher values indicating better discriminatory test performance. A DOR of 1 indicates that the test does not discriminate between those with improved and those with worsened anchor response. Figures are presented with descriptive plots of mean values with CI. Non-overlapping CIs were interpreted as significant differences. Statistical analyses were performed using the software package IBM SPSS Statistics for Windows, Version 29 (IBM Corp, Armonk, NY, USA).

### Ethics, funding, and disclosures

The study was approved by the regional committee for medical and health research ethics (REC central) (approval no.656585). All methods were carried out in accordance with the relevant guidelines and regulations. Patients were informed about the registry and gave written informed consent to allow data to be used for scientific purposes before they were included. The study did not receive any grant or funding, and the authors declare no conflicts of interests. Complete disclosure of interest forms according to ICMJE are available on the article page, doi: 10.2340/17453674.2024.42098

## Results

Of 3,243 cases, 2,478 were included after exclusion of revisions within 1 year and cases with missing KOOS-PS score or answer to the anchor question (Figure 1). Baseline patient characteristics were similar among the groups with an average of 67 years, a BMI of 29 and approximately 60% women (Table).

### KOOS-PS

The analysis showed agreement between the anchor question and the KOOS-PS with a DOR of 11.3 (7.9–16.2). The diagnostic test accuracy found a positive predictive value (PPV) of 91%, where 2,132 of the 2,335 patients who reported improved function on the anchor question had improved KOOS-PS at 1-year follow-up. Of the remaining 143 who reported worsened function on the anchor question 74 had worsened KOOS-PS (WW), a negative predicted value (NPV) of 52% (Figure 2).

The test showed a sensitivity of 97%, whereby 2,132 of 2,201 patients who had improved KOOS-PS at 1-year follow-up reported improved function on the anchor question. The test specificity was 27%, as 74 of those 277 with a worsened KOOS-PS (WW) reported worsened function on the anchor question (Figure 2).

**Figure 3:**
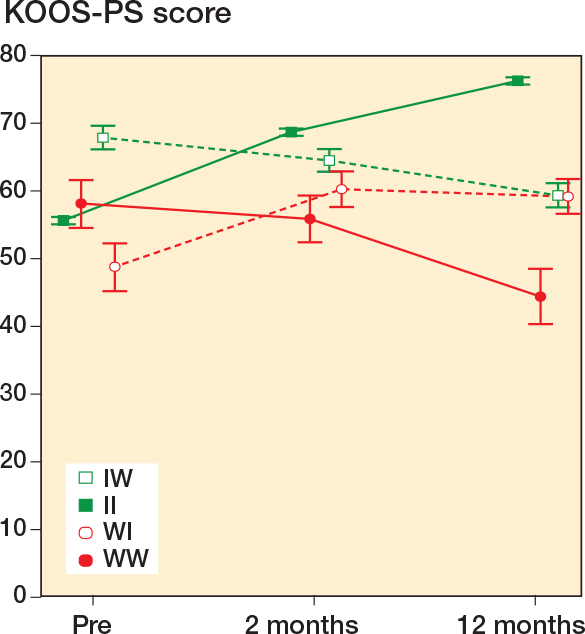
Descriptive values of KOOS-PS preoperatively, and at 2-month and 1-year follow-up in the 4 TKA groups dichotomized by the anchor question, and accordingly change in KOOS-PS. For abbreviations, see Legend to Figure 2.

**Figure 4:**
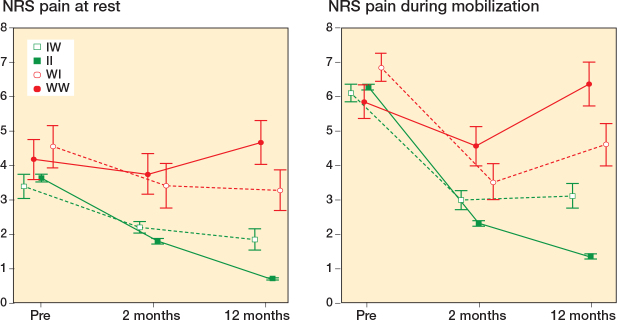
Descriptive values of pain at rest and pain during mobilization preoperatively, at 2-month and 1-year follow-up in the 4 TKA groups dichotomized by the anchor question, and accordingly change in KOOS-PS. For abbreviations, see Legend to Figure 2.

Consistency between the response on the anchor question and change in KOOS-PS was found in 2,206/2,478 (89%) of the cases (II and WW). These 2 consistency groups had the best and worse results in KOOS-PS at 1-year follow-up (Figures 3 and 4). Inconsistency was found in 272/2,478 (11%) of the cases (IW and WI), in which KOOS-PS from preoperatively until 1-year follow-up was statistically significant worsened and improved, respectively. These groups had similar KOOS-PS at 1-year follow-up (Figure 3).

### Pain

Statistically significant differences in pain at rest and during mobilization were found between all 4 groups at 1-year follow-up. The 2 groups who reported improved anchor (II and IW) demonstrated less pain than the 2 groups with worsened anchor (WW and WI). The II group had least pain at 1-year follow-up, followed by the IW, the WI, and finally the WW group, who had most pain both at rest and during mobilization (Figure 4).

Surgery time, LOS, and complications are presented in the Table.

## Discussion

We aimed to evaluate whether a simple anchor question can replace KOOS-PS in assessing postoperative knee function until 1-year follow-up after primary TKA. We showed that at 1-year follow-up of patients who reported improved anchor, 91% had improved KOOS-PS whereas among patients reporting worsened function only 52% had worsened KOOS-PS.

The 2 groups with consistency between the anchor question and the KOOS-PS had the best and worst results at each follow-up, on all outcomes (Figures 3 and 4). The 2 groups with inconsistency had similar KOOS-PS at 1-year follow-up; however, the IW group have less pain than the WI group, which indicates that pain is important for the patient’s response on the anchor question.

Pain has previously been found to be the most important determinant for patient satisfaction, and postoperative scores have demonstrated higher correlation with patient satisfaction as compared with preoperative scores or changes in scores (6). Low pain levels at 1 year could explain why patients report improved function on the anchor question despite a worsened KOOS-PS (IW) in our study. Likewise, patients with worsened anchor when the score is improved (WI) have higher pain at 1 year. Our results demonstrating that patients who report improved function have less pain than patients reporting worsened function, regardless of the change in KOOS-PS, indicate that patients not only assess the change in function based on preoperative results, but also related to their current sensation of pain. Pain and function are decisive for patient satisfaction [16], which supports our findings suggesting that our anchor question asking the patients about change in function is a reliable tool in evaluating overall patient satisfaction.

KOOS-PS has been recommended as a measure of TKA outcome [17]. However, it has been found that it might not adequately reflect physical functioning [18]. This partly supports our findings demonstrating that KOOS-PS might not be a reliable tool alone in the evaluation of the change in function at 1-year follow-up as there are inconsistencies between the anchor response and the KOOS-PS, especially among patients reporting worsened anchor. The anchor question asks the patients about their impression of knee function related to preoperative function and ideally, if the question was able to reveal the true function, the responses should be consistent with the change in KOOS-PS. This was not the case in our study among patients reporting worsened function and shows that other factors such as pain are decisive for the patient’s response on the anchor question.

Previous studies have found that patients do not recall their preoperative function very well [12] and that their memories of preoperative pain and function are inaccurate beyond 3 months after TKA [11,12,19]. This could explain our findings of inconsistency between the anchor question and the KOOS-PS in some patients. The fulfillment of preoperative expectations concerning improvement in knee function and pain relief influences the patient’s assessment of the outcome [2,4,20-23], and is significantly associated with the level of satisfaction at 1 year [3,24]. Met expectations haves even been found to moderate the relationship between pain and satisfaction [4]. Patients may be overly optimistic concerning the chance of being pain free and unlimited in physical activities [25], which are the most important contributing factors for patient satisfaction [1,16,25,26]. Therefore, it seems important to clarify expectations regarding the results preoperatively so that they are realistic and achievable.

Our results demonstrate high agreement between the anchor question and the KOOS-PS, which implies that the anchor question can replace the use of the KOOS-PS. Nevertheless, almost half of the patients reporting worse function on the anchor question in the present study had improved KOOS-PS. The experience of worsened function in these patients is attributed to the higher level of pain, which is supported by others demonstrating pain to be an important contributor to impaired function after TKA [27]. Our findings indicate that the anchor question could probably be supplemented by the KOOS-PS to reveal the change in function in patients reporting worse function.

The advantages of using a simple anchor question are to ease the question burden for the patients and the healthcare providers, and to evaluate the outcome in cases where there are no preoperative PROMs available. The level of completeness of the PROMs is of great importance for the reliability of the results [28]. Replacing the score with an anchor question simplifies the process and might therefore improve the reporting. However, the KOOS-PS constitutes of only 7 items in contrast to the 40 items of the full KOOS and should be manageable for most patients [29]. The response on the anchor question is appropriate in evaluating change of function at an individual level but in populations there might be some advantages of using the KOOS-PS. Subjective information is transformed into quantitative measures, and gives a score which enables assessment of the level of function [30]. When obtained repeatedly it gives the possibility to evaluate change over time, and it is also more detailed than the anchor question and thereby it could detect minor differences [17,31].

Some studies report that patient characteristics, preoperative pain, and PROM scores are predictive for clinical outcomes after TKA [1,32,33], but not at a level that is clinically useful [34]. In our study, the preoperative patient characteristics among the groups were similar and only some minor variations were shown in preoperative pain (Figure 4). The consistency groups had about the same preoperative KOOS-PS score but differed by more than 30 points at 1 year. In contrary, the inconsistency groups started with a 20-point difference preoperatively but ended up with similar scores at 1 year (Figure 3). The inconsistency groups similarly and significantly improved pain at rest and during mobilization from preoperatively to 1-year follow-up, but those who reported improved anchor despite worsened KOOS-PS had significantly less pain at all follow-ups.

The strength of the study is the high number of patients included, in which all were attending a standardized Fast-track clinical setting. An important part of Fast-track is extensive preoperative information provided in a preoperative education class, where the patients receive the same information regarding anticipated outcomes [15]. Even though similar information is provided to all patients, the perception and how this information is absorbed in each patient differs, which probably results in dissimilar expectations.

### Limitations

The anchor question has not been tested with cognitive interviews or focus groups. Neither do we have any information concerning the patient’s preoperative expectations which might influence their responses.

### Conclusion

We showed a high agreement between the anchor question and the KOOS-PS and demonstrated that the KOOS-PS can be replaced with an anchor question to assess change in function after primary TKA. However, the KOOS-PS might be a valuable supplement in patients reporting worsened anchor as only half of those had worsened KOOS-PS. The patient’s response on the anchor question is influenced by the level of pain at 1 year.

## Notes

SBW and JK planned the study and interpretated the data. AS and TSW performed surgeries. SLS and SBW contributed to data registration. JK performed the data analyzes. All authors contributed to revising the draft manuscript written by SBW.

Handling co-editors: Li Felländer-Tsai and Robin Christensen

*Acta* thanks Eva Broström and Bart G Pijls for help with peer review of this manuscript.
